# Efficacy and safety of capecitabine plus cisplatin in Japanese patients with advanced or metastatic gastric cancer: subset analyses of the AVAGAST study and the ToGA study

**DOI:** 10.1007/s10120-012-0167-0

**Published:** 2012-07-11

**Authors:** Kensei Yamaguchi, Akira Sawaki, Toshihiko Doi, Taroh Satoh, Yasuhide Yamada, Yasushi Omuro, Tomohiro Nishina, Narikazu Boku, Keisho Chin, Yasuo Hamamoto, Hiroya Takiuchi, Yoshito Komatsu, Shigehira Saji, Wasaburo Koizumi, Yoshinori Miyata, Atsushi Sato, Eishi Baba, Takao Tamura, Takashi Abe, Atsushi Ohtsu

**Affiliations:** 1 Department of Gastroenterology, Saitama Cancer Center Hospital, 818 Komuro, Ina-machi, Kitaadachi-gun, Saitama, 362-0806 Japan; 2 Department of Gastroenterology, Aichi Cancer Center Hospital, Aichi, Japan; 3 Gastrointestinal Oncology Division, National Cancer Center Hospital East, Chiba, Japan; 4 Department of Medical Oncology, Kinki University School of Medicine, Osaka, Japan; 5 Department of Gastrointestinal Oncology, National Cancer Center Hospital, Tokyo, Japan; 6 Department of Chemotherapy, Tokyo Metropolitan Cancer and Infectious Diseases Center, Komagome Hospital, Tokyo, Japan; 7 Department of Internal Medicine, National Hospital Organization Shikoku Cancer Center, Ehime, Japan; 8 Department of Gastroenterology, Shizuoka Cancer Center, Shizuoka, Japan; 9 Department of Gastroenterology, JFCR Cancer Institute Ariake Hospital, Tokyo, Japan; 10 Department of Medical Oncology, Tochigi Cancer Center, Tochigi, Japan; 11 Cancer Chemotherapy Center, Osaka Medical College, Osaka, Japan; 12 Third Department of Internal Medicine, Hokkaido University Hospital, Hokkaido, Japan; 13 Department of Medical Oncology, Saitama Medical University International Medical Center, Saitama, Japan; 14 Department of Gastroenterology, Kitasato University East Hospital, Kanagawa, Japan; 15 Department of Gastroenterology, Saku Central Hospital, Nagano, Japan; 16 Department of Internal Medicine, Toyosu Hospital, Showa University School of Medicine, Tokyo, Japan; 17 Department of Hematology and Oncology, Kyushu University Hospital, Fukuoka, Japan; 18 Division of Diabetes, Digestive, and Kidney Diseases, Department of Clinical Molecular Medicine, Kobe University Graduate School of Medicine, Hyogo, Japan; 19 Internal Medicine, Yamagata Prefectural Central Hospital, Yamagata, Japan; 20 Research Center for Innovative Oncology, National Cancer Center Hospital East, Chiba, Japan

**Keywords:** Capecitabine, Cisplatin, Gastric cancer, Japanese patients, Subset analysis

## Abstract

**Background:**

Capecitabine plus cisplatin (XP) is recognized as one of the global standard first-line chemotherapy regimens for patients with metastatic gastric cancer (mGC). Recent multinational phase III trials in mGC have been conducted with XP as the control arm, although no data on XP in Japanese patients with mGC have been published to date. The AVAGAST (XP ± bevacizumab in mGC) and ToGA (XP ± trastuzumab in human epidermal growth factor receptor 2 [HER2]-positive mGC) studies were the first two global studies including Japanese mGC patients. The aim of this analysis was to investigate the efficacy and safety of XP in Japanese mGC patients, using AVAGAST and ToGA subgroup data.

**Methods:**

Efficacy and safety analyses were carried out in Japanese patients with mGC receiving XP alone, based on results from the AVAGAST and ToGA studies. There were differences in the target populations between the two studies; for example, the ToGA study limited patients to those with HER2-positive tumors; therefore, efficacy was evaluated separately.

**Results:**

Ninety-four Japanese patients in the AVAGAST study and 50 in the ToGA study received XP alone. Median overall and progression-free survivals were 14.2 and 5.7 months, respectively, in the AVAGAST study, and 17.7 and 5.6 months, respectively, in the ToGA study. Overall response rates were 49.2 % in the AVAGAST and 58.5 % in the ToGA study. Adverse events were generally mild; the most common grade 3/4 events were neutropenia, anemia, anorexia, and nausea.

**Conclusions:**

XP is effective and well tolerated in Japanese patients with mGC, and could be one of the standard regimens for the first-line treatment in this cohort.

## Introduction

Gastric cancer remains one of the most common forms of cancer worldwide, with an incidence of approximately 870,000 new cases per year and 650,000 deaths per year [1, 2], accounting for about 9.9 % of new cancers [3]. In Japan, the incidence of gastric cancer is around 110,000 new cases each year, with around 50,000 deaths reported in 2009 [4]. Chemotherapy is the most effective treatment for patients with unresectable advanced and metastatic gastric cancer (mGC). The combination of a fluoropyrimidine (5-fluorouracil or an oral fluoropyrimidine) plus cisplatin has been one of the most commonly used regimens due to its activity and well-established toxicity profile.

Capecitabine is an oral fluoropyrimidine that undergoes a three-step enzymatic activation process, the last step of which occurs selectively within the tumor tissue itself. The comparable efficacy of regimens substituting capecitabine for infused 5-flurouracil has been directly studied in two phase III trials: the REAL-2 study and the ML17032 study. A meta-analysis of these two trials concluded that, compared with 5-flurouracil combinations, capecitabine combinations were associated with higher response rates (odds ratio 1.38, 95 % confidence interval [CI] 1.10–1.73) and better overall survival (hazard ratio for death 0.87, 95 % CI 0.77–0.98) [5]. The combination of capecitabine and cisplatin (XP) is therefore recognized worldwide as one of the standard first-line chemotherapy regimens for patients with mGC.

More recent phase III studies, including the AVAGAST, ToGA, and EXPAND studies, have focused on the benefit of adding a molecular targeting agent to the XP regimen (bevacizumab, trastuzumab, and cetuximab in each of these studies, respectively). The AVAGAST and ToGA studies (conducted in human epidermal growth factor receptor 2 [HER2]-positive mGC patients) were the first two global studies including Japanese patients with mGC. Japanese patients comprised 94 of 387 patients receiving XP alone in the AVAGAST study, and 50 of 290 patients receiving XP alone in the ToGA study.

In the Japanese *Gastric cancer treatment guideline (3rd edition)* [6], S-1 (a compound preparation containing the oral fluoropyrimidine tegafur) in combination with cisplatin (SP) is recommended as a standard first-line chemotherapy regimen in Japan. However, there has been no efficacy or safety information on the XP regimen for the Japanese population.

The aim of this analysis was to investigate the efficacy and safety of XP in Japanese patients with mGC using subgroup data from the AVAGAST study and the ToGA study. There were differences in the target populations in the two studies; for example, the ToGA study was limited to patients with tumors showing overexpression of HER2. Because it was not known whether the efficacy of chemotherapy differed between HER2-positive and -negative tumors, we evaluated efficacy in the ToGA and AVAGAST studies separately. For adverse events, we combined the data from the ToGA and AVAGAST studies.

## Patients and methods

### Patients

The main eligibility criteria common to both the studies above were as follows: metastatic or inoperable locally advanced adenocarcinoma of the stomach or gastro-esophageal junction; measurable (according to response evaluation criteria in solid tumors [RECIST]) or evaluable disease; adequate organ function; Eastern Cooperative Oncology Group (ECOG) performance status 0–2; written informed consent; creatinine clearance ≥60 mL/min; no previous adjuvant chemotherapy within 6 months; no previous chemotherapy for metastatic or locally advanced gastric cancer; and no history of other malignancies.

The ToGA study limited patients to those with HER2-positive tumors (centrally assessed, immunohistochemistry [IHC] 3+ and/or fluorescent in situ hybridization [FISH] +) [7], no congestive heart failure, and baseline left ventricular ejection fraction (LVEF) ≥50 %. In the AVAGAST study, the exclusion criteria included uncontrolled hypertension or clinically significant (i.e., active) cardiovascular disease.

### Treatment

The AVAGAST study was a randomized double-blind placebo-controlled study, and the ToGA study was an open-label randomized controlled study. Patients were randomly assigned in a 1:1 ratio to receive monoclonal antibody therapy (bevacizumab in AVAGAST; trastuzumab in ToGA) plus chemotherapy (capecitabine or fluorouracil plus cisplatin), or chemotherapy alone.

The chemotherapy regimen for Japanese patients was exclusively XP in both studies. Cisplatin 80 mg/m^2^ was given by intravenous infusion on day 1 of each 3-week cycle. Capecitabine 1,000 mg/m^2^ was given orally twice a day on days 1–14 of the cycle followed by a 7-day rest period. XP doses were modified according to the hematological toxicities on the first day of a planned course (Table 1). If the absolute neutrophil count (ANC) was <1 × 10^9^/L and the platelet count was <100 × 10^9^/L, treatment was delayed; and then if hematological parameters did not recover within 3 weeks after the delay, treatment was discontinued. Cisplatin doses were also modified to account for renal function, as shown in Table 2.

**Table 1:** Dose modification for hematological toxicity on the first day of a planned course

ANC and platelet count	Capecitabine/cisplatin dose modification
≥1.5 × 10^9^ and ≥100 × 10^9^/L	100 % of original dose of capecitabine and cisplatin given without a delay
≥1 − <1.5 × 10^9^ and ≥100 × 10^9^/L	75 % of original dose of capecitabine and cisplatin given without a delay
<1 × 10^9^ and/or <100 × 10^9^/L	Delay until recovery to ANC ≥1 × 10^9^/L and platelets ≥100 × 10^9^/L. Then, if ANC ≥1 − <1.5 × 10^9^/L, re-commence at 75 % of original dose of capecitabine and cisplatin. Thereafter in post-dose reduction treatment courses, if ANC ≥1.5 × 10^9^/L, then re-commence at 100 % of original dose of capecitabine and cisplatin

*ANC* absolute neutrophil count

**Table 2:** Dose modification of cisplatin in renal impairment

Creatinine clearance (mL/min)	AVAGAST	ToGA
≥60	100 % of dose	100 % of dose
51–59	Reduce dose by 25 %	Same dose of cisplatin in mg/m^2^ as the value of the Ccr in mL/min
41–50	Reduce dose by 50 %	
≤40	Stop cisplatin permanently	Stop cisplatin permanently

Creatinine clearance (Ccr) was determined mainly by the Cockcroft and Gault calculation. If Ccr was evaluated by the direct method then dose modification was based on this

### Assessment of response and toxicity

In both studies the primary endpoint was overall survival. Secondary endpoints included progression-free survival, time to progression, overall tumor response rate, clinical benefit rate (defined as patients without progressive disease in AVAGAST; and patients with best overall response of confirmed complete response, partial response, or stable disease in ToGA), duration of response, and safety. These endpoints were based on regular assessments of disease response and progression using RECIST criteria. In AVAGAST, tumor assessments were performed every 6 weeks for the first year after randomization and thereafter every 12 weeks until disease progression. In ToGA, tumor assessments were performed every 6 weeks until disease progression.

Adverse events were assessed according to the National Cancer Institute Common Terminology Criteria for Adverse Events (NCI-CTCAE), version 3.0.

### Statistical analysis

The analyses of overall survival and progression-free survival were based on the survival analysis; median time was estimated using the Kaplan–Meier method, the 95 % confidence interval (CI) for the median was calculated by the Brookmeyer-Crowley method. Overall tumor response rate was defined as the proportion of occurrence of either a confirmed complete or a partial best overall response.

Dose intensity was defined as the actual dose administered divided by the planned dose. In the AVAGAST study the planned dose of cisplatin was the entire dose of all six cycles, and that of capecitabine was the whole dose of an actual administered cycle. In the ToGA study the planned dose was the whole dose of an actual administered cycle.

## Results

### Patient characteristics

In the AVAGAST study between September 2007 and December 2008, a total of 774 patients were enrolled at 93 centers in 17 countries. There were 188 Japanese patients enrolled at 14 centers, with 94 patients in the XP group. In the ToGA study between September 2005 and December 2008, a total of 594 patients were enrolled at 122 centers in 24 countries; 102 patients were enrolled at 16 centers in Japan. One patient was excluded from the analysis as they received no treatment, resulting in a total of 101 patients being the subjects for the analysis, with 50 patients in the XP group.

Table 3 shows the demographics and baseline characteristics of the cohorts. Median follow-up times were 12.0 months (0.1–23.9 months) in the AVAGAST study and 17.1 months (1–49 months) in the ToGA study.

**Table 3:** Baseline patient characteristics

	AVAGAST^a^ (*N* = 94)	ToGA^b^ (*N* = 50)
Sex (%)		
Male	63 (67.0)	40 (80.0)
Female	31 (33.0)	10 (20.0)
Median age		
Years (range)	61.0 (36–78)	63.5 (45–81)
Extent of disease (%)		
Locally advanced	1 (1.1)	1 (2.0)
Metastatic	93 (98.9)	49 (98.0)
Primary tumor site (%)		
Stomach	88 (93.6)	44 (88.0)
Gastro-esophageal junction	6 (6.4)	6 (12.0)
Measurability of disease (%)		
Measurable	65 (69.1)	41 (82.0)
Non-measurable	29 (30.9)	9 (18.0)
ECOG performance status (%)		
0–1	94 (100.0)	50 (100.0)
Number of metastatic sites at baseline (%)		
0	1 (1.1)	–
1	34 (36.2)	–
≥2	59 (62.8)	–
1–2	–	32 (64.0)
>2	–	18 (36.0)
Type of gastric cancer^c^ (%)		
Intestinal type	22 (23.4)	42 (84.0)
Diffuse type	65 (69.1)	4 (8.0)
Mixed type	7 (7.4)	4 (8.0)
Visceral metastasis (%)		
Liver metastasis	23 (24.5)	–
Liver or lung metastasis	–	33 (66.0)
History of treatment for gastric cancer (%)		
Prior gastrectomy	31 (33.0)	13 (26.0)
Prior chemotherapy	8 (8.5)	0

^a^The target population of the AVAGAST study was metastatic or inoperable locally advanced adenocarcinoma of the stomach or gastro-esophageal junction

^b^The target population of the ToGA study was human epidermal growth factor receptor 2 (HER2)-positive metastatic or inoperable locally advanced adenocarcinoma of the stomach or gastro-esophageal junction

^c^The type of gastric cancer is as described in the Lauren classification

### Efficacy

**Fig. 1:**
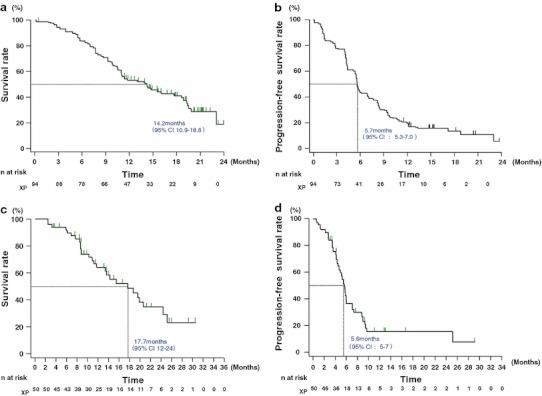
**a** AVAGAST: overall survival. Median overall survival was 14.2 months (95 % confidence interval [*CI*], 10.9–18.8 months). **b** AVAGAST: progression-free survival. Median progression-free survival was 5.7 months (95 % CI, 5.3–7.0 months). **c** ToGA: overall survival. Median overall survival was 17.7 months (95 % CI, 12–24 months). **d** ToGA: progression-free survival. Median progression-free survival was 5.6 months (95 % CI, 5–7 months)

In the AVAGAST study, the median overall survival was 14.2 months (95 % CI, 10.9–18.8 months; Fig. 1a) and median progression-free survival was 5.7 months (95 % CI, 5.3–7.0 months; Fig. 1b). Patients with measurable disease comprised 65 of the 94 patients. The overall tumor response rate was 49.2 % (32/65 patients) and the clinical benefit rate was 67.7 % (44/65 patients). Median time to progression was 5.6 months (95 % CI, 5.1–7.2 months) and median response duration was 6.9 months (95 % CI, 4.2–9.5 months; Table 4).

**Table 4:** Analysis of efficacy

Endpoints	AVAGAST^a^ (*N* = 94)	ToGA^b^ (*N* = 50)
Median overall survival (months) (95 % CI)	14.2 (10.9–18.8)	17.7 (12–24)
Median progression-free survival (months) (95 % CI)	5.7 (5.3–7.0)	5.6 (5–7)
Median time to progression (months) (95 % CI)	5.6 (5.1–7.2)	5.6 (5–7)
Response rate^c^ (%)	49.2 (32/65)	58.5 (24/41)
Clinical benefit rate^c^ (%)	67.7 (44/65)	85.4 (35/41)
Median response duration (months) (95 % CI)	6.9 (4.2–9.5)	4.3 (4–7)

*95* *% CI* 95 % confidence interval

^a^The target population of the AVAGAST study was metastatic or inoperable locally advanced adenocarcinoma of the stomach or gastro-esophageal junction

^b^The target population of the ToGA study was HER2-positive metastatic or inoperable locally advanced adenocarcinoma of the stomach or gastro-esophageal junction (GEJ)

^c^Measurable disease

In the ToGA study, the median overall survival was 17.7 months (95 % CI, 12.0–24.0 months; Fig. 1c) and the median progression-free survival was 5.6 months (95 % CI, 5.0–7.0 months; Fig. 1d). Patients with measurable disease comprised 41 of the 50 patients. The overall tumor response rate was 58.5 % (24/41 patients), and the clinical benefit rate was 85.4 % (35/41 patients). Median time to progression was 5.6 months (95 % CI, 5.0–7.0 months) and median response duration was 4.3 months (95 % CI, 4.0–7.0 months; Table 4).

### Safety

In the AVAGAST study, capecitabine exposure was as follows: the median number of treatment cycles was 7; the median total dose received was 243,900 mg; and the median dose intensity was 80 %. Cisplatin exposure was as follows: the median number of treatment cycles was 6; the median total dose received was 539 mg; and the median dose intensity was 71 %.

In the ToGA study, capecitabine exposure was as follows: the median number of treatment cycles was 6; the median total dose received was 210,000 mg; and the median dose intensity was 84 %. Cisplatin exposure was as follows: the median number of treatment cycles was 6; the median total dose received was 569 mg; and the median dose intensity was 83.3 %.

Grade 3 or 4 adverse events occurred in 107 (74 %) patients and those events occurring at a frequency of greater than 10 % were as follows: neutropenia 45 %, anorexia 26 %, nausea 17 %, and anemia 13 %. Grade 3 or 4 diarrhea (4 %) and hand-foot syndrome (2 %) occurred at much lower frequencies compared with the incidences of each event in all grades. A full summary of the adverse events is shown in Table 5. Treatment was terminated due to adverse events for 12 (13 %) patients in the AVAGAST study and 4 (8 %) patients in the ToGA study. The type and severity of adverse events were consistent with previous reports of these drugs. There were no treatment-related deaths. In the AVAGAST study, the median time to initial onset of the first episode of neutropenia was 1.4 months (the start of cycle 3); for renal impairment it was 21 days (the start of cycle 2); and for nausea or vomiting it was 3 days (after the administration of cisplatin in cycle 1).

**Table 5:** Summary of adverse events

	AVAGAST^a^ (*N* = 94)		ToGA^b^ (*N* = 50)		Total (*N* = 144)	
	All grades	Grade 3	All grades	Grade 3	All grades	Grade 3
	*N* (%)	*N* (%)	*N* (%)	*N* (%)	*N* (%)	*N* (%)
Total	94 (100)	71 (76)	50 (100)	36 (72)	144 (100)	107 (74)
Hematological toxicities						
Neutropenia	63 (67)	45 (48)	34 (68)	20 (40)	97 (67)	65 (45)
Thrombocytopenia	19 (20)	2 (2)	8 (16)	3 (6)	27 (19)	5 (3)
Anemia	16 (17)	10 (11)	11 (22)	8 (16)	27 (19)	18 (13)
Febrile neutropenia	5 (5)	5 (5)	3 (6)	3 (6)	8 (6)	8 (6)
Non-hematological toxicities						
Nausea	84 (89)	18 (19)	44 (88)	7 (14)	128 (89)	25 (17)
Vomiting	60 (64)	6 (6)	28 (56)	2 (4)	88 (61)	8 (6)
Diarrhea	51 (54)	4 (4)	24 (48)	2 (4)	75 (52)	6 (4)
Stomatitis	34 (36)	1 (1)	16 (32)	1 (2)	50 (35)	2 (1)
Abdominal pain	12 (13)	1 (1)	3 (6)	–	15 (10)	1 (<1)
Hand-foot syndrome	54 (57)	2 (2)	23 (46)	1 (2)	77 (53)	3 (2)
Rash	19 (20)	–	5 (10)	–	24 (17)	–
Anorexia	83 (88)	27 (29)	46 (92)	10 (20)	129 (90)	37 (26)
Fatigue	69 (73)	5 (5)	26 (52)	4 (8)	95 (66)	9 (6)
Peripheral neuropathy	28 (30)	2 (2)	10 (20)	–	38 (26)	2 (1)
Renal impairment	17 (18)	3 (3)	27 (54)	–	44 (31)	3 (2)
Increased lacrimation	2 (2)	–	1 (2)	–	3 (2)	–

^a^The target population of the AVAGAST study was metastatic or inoperable locally advanced adenocarcinoma of the stomach or gastro-esophageal junction

^b^The target population of the ToGA study was HER2-positive metastatic or inoperable locally advanced adenocarcinoma of the stomach or gastro-esophageal junction

## Discussion

In our analysis of the efficacy of XP, comparing the results from the XP group of Japanese subjects in the AVAGAST study with those of the overall chemotherapy group [8] showed that median progression-free survival times (5.7 vs. 5.3 months) were equivalent, but the median survival time (14.2 vs. 10.1 months) was longer for Japanese subjects. A similar comparison of the results from the XP group of Japanese subjects in the ToGA study with those of the overall chemotherapy group [7] also showed median progression-free survival times (5.6 vs. 5.5 months) to be equivalent, with median survival times of 17.7 vs. 11.1 months; again, appreciably longer in Japanese subjects. In Japanese patients, the second-line regimen, after the failure of the first-line treatment, was usually irinotecan- or taxane-based [9, 10], which might have led to the favorable results in two Japanese studies.

Safety analysis demonstrated similar tolerability in the XP group of Japanese subjects to that of the overall chemotherapy group. In the AVAGAST study, this was shown by the median dose intensity of capecitabine (80.0 vs. 87.0 %), median dose intensity of cisplatin (71.0 vs. 71.0 %), and the incidence of grade 3 or higher adverse events (76 vs. 77 %), and in the ToGA study by the median dose intensity of capecitabine (84.0 vs. 86.7 %), the median dose intensity of cisplatin (83.3 vs. 91.1 %), and the incidence of grade 3 or higher adverse events (72 vs. 68 %). These results provide ample evidence of the efficacy and tolerability of XP in Japanese patients.

Comparing the Japanese XP groups in the AVAGAST and ToGA studies, the incidence of intestinal-type gastric cancer in the ToGA study (which limited patients to HER2-positive tumors only) was higher than that in the AVAGAST study (84.0 vs. 23.4 %). The incidence of non-measurable disease in the AVAGAST study was higher than that in the ToGA study (30.9 vs. 18.0 %) because patients with peritoneal disease, which could be diagnosed by laparoscopy or laparotomy, were allowed to enter the AVAGAST study, as they were generally regarded as having evaluable disease. In the analysis of efficacy, median progression-free survival was equivalent (AVAGAST, 5.7 months vs. ToGA, 5.6 months). However, median survival time was longer in the ToGA study (AVAGAST, 14.2 months vs. ToGA, 17.7 months) although the reason for this is unknown. The profiles and frequencies of adverse events were similar in both studies. The follow-up period of the AVAGAST study was shorter than that of the ToGA study and further follow up might be necessary.

The dose modification methods used for cisplatin in patients who demonstrated decreased creatinine clearance were different in the AVAGAST and ToGA studies (Table 2). However, given that there were no major differences between the AVAGAST study and the ToGA study in terms of efficacy, safety, or the percentage of patients with cisplatin dose reduction up to cycle 6 in the Japanese XP group, and considering the fact that the dose modification method for cisplatin in the AVAGAST study is simpler, the dose modification method for cisplatin in the AVAGAST study is recommended to be adopted as the standard approach for XP in Japan.

It is also important to note that the durations of treatment with capecitabine were different in the AVAGAST and ToGA studies. In the AVAGAST study capecitabine could be continued until disease progression, but in the ToGA study it could be continued initially up to cycle 6. After July 2007, owing to a protocol amendment in the second half of the study, capecitabine could subsequently be continued until disease progression. Although maintenance treatment with capecitabine has been proven effective in colorectal cancer, it has not been studied in mGC. Debate continues on the effectiveness of maintenance therapy in gastric cancer and further investigations are required before this becomes common practice.

Based on the results of the JCOG 9912 study [11] and SPIRITS study [12], the Japanese *Gastric cancer treatment guideline (3rd edition)* [6] recommends the combination of S-1 plus cisplatin (SP) as the standard first-line chemotherapy for unresectable advanced or mGC. A comparison of the SP cohort in the SPIRITS study [12] and the Japanese XP cohort in the AVAGAST study showed their main clinical characteristics to be similar. In the analysis of efficacy, the median survival time was 13.0 months for SP and 14.2 months for XP. The 1-year survival rate was 54.1 % for SP and 53.7 % for XP, demonstrating that the results for the Japanese XP group were not inferior to those for SP. Grade 3/4 adverse events (incidence of ≥10 %) were neutropenia, anorexia, anemia, leucopenia, and nausea for SP, and the events and frequencies were similar in the Japanese XP group. There were also no major differences in the frequencies of grade 3/4 diarrhea or hand-foot syndrome.

Considering that the global standard for patients with mGC is combination therapy with a fluoropyrimidine plus a platinum compound, it is possible that XP could be ranked alongside SP as one of the standard regimens in the first-line treatment of unresectable advanced or mGC in Japan. However, capecitabine and S-1 are of different drug designs and it has been reported that predictive factors for the efficacy of these two agents are different [13, 14]. It is anticipated that further research will establish the appropriate use of each fluoropyrimidine, using biomarkers and other measures.

By adjusting the dose according to the criteria for dose modification and discontinuation (Tables 1, 2), the XP regimen permits the continuation of treatment when adverse reactions occur. It will be important to know which adverse reactions may occur, and when. In the Japanese XP cohort of the AVAGAST study the main adverse events that led to interruption, dose modification, or discontinuation of capecitabine or cisplatin included neutropenia, renal impairment, and nausea or vomiting. In the AVAGAST study, the median time to the initial onset of the first episode of neutropenia was 1.4 months (the start of cycle 3); for renal impairment it was 21 days (the start of cycle 2); and for nausea or vomiting it was 3 days (after the administration of cisplatin in cycle 1).

**Fig. 2:**
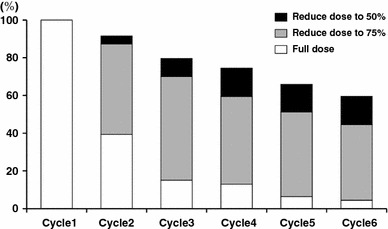
AVAGAST: situation of cisplatin dose reduction by treatment cycle. The dose of cisplatin was reduced from 80 to 60 mg/m^2^ at cycle 2 in about 50 % of the patients. Cisplatin therapy was continued even at cycle 6

For Japanese patients, no phase I trial combining XP in a 3-week cycle has been conducted. In the AVAGAST study, dose modification of capecitabine due to adverse events was reported in 83 patients (88.3 %), but discontinuation due to adverse events occurred in only 6 patients (6 %). The starting dose of cisplatin was 80 mg/m^2^, but, as illustrated in the analysis in Fig. 2, about 50 % of the patients required a dose reduction in the second cycle. It is considered that there might be a more feasible dosing schedule for Japanese patients. Dose reduction of cisplatin due to adverse events (including laboratory abnormalities) was reported in 75 patients (79.8 %), but discontinuation due to adverse events was necessary in just 7 patients (7 %). Treatment was continued by adjusting the dose in such patients. Given the above observations, it will be important to cautiously monitor the adverse events that are the main causes of dose modification, i.e., neutropenia, renal impairment, and nausea or vomiting, and appropriately adjust the dose up to the time at which cisplatin is given in combination, and in particular at least until the third cycle after starting XP treatment for gastric cancer.

The present research is the first analysis focusing on the efficacy and safety of XP in Japanese patients with advanced gastric cancer. Based on the results of this study, XP is considered to be acceptable as a standard control arm in Japanese patients. Furthermore, by modifying the doses of XP to manage adverse reactions, XP could be one of the standard regimens for the first-line treatment of unresectable advanced or recurrent gastric cancer in Japan.
